# Unraveling the Impact of Cysteine-to-Serine Mutations on the Structural and Functional Properties of Cu(I)-Binding Proteins

**DOI:** 10.3390/ijms20143462

**Published:** 2019-07-14

**Authors:** Matic Pavlin, Zena Qasem, Hila Sameach, Lada Gevorkyan-Airapetov, Ida Ritacco, Sharon Ruthstein, Alessandra Magistrato

**Affiliations:** 1 CNR-IOM at SISSA, via Bonomea 265, 34135 Trieste, Italy; 2 Department of Chemistry, Faculty of Exact Sciences, Bar Ilan University, Ramat-Gan 5290002, Israel

**Keywords:** copper metabolism, serine mutations, Cu(I) affinity, CueR, Atox1, ATP7B, EPR, molecular dynamics, QM/MM

## Abstract

Appropriate maintenance of Cu(I) homeostasis is an essential requirement for proper cell function because its misregulation induces the onset of major human diseases and mortality. For this reason, several research efforts have been devoted to dissecting the inner working mechanism of Cu(I)-binding proteins and transporters. A commonly adopted strategy relies on mutations of cysteine residues, for which Cu(I) has an exquisite complementarity, to serines. Nevertheless, in spite of the similarity between these two amino acids, the structural and functional impact of serine mutations on Cu(I)-binding biomolecules remains unclear. Here, we applied various biochemical and biophysical methods, together with all-atom simulations, to investigate the effect of these mutations on the stability, structure, and aggregation propensity of Cu(I)-binding proteins, as well as their interaction with specific partner proteins. Among Cu(I)-binding biomolecules, we focused on the eukaryotic Atox1-ATP7B system, and the prokaryotic CueR metalloregulator. Our results reveal that proteins containing cysteine-to-serine mutations can still bind Cu(I) ions; however, this alters their stability and aggregation propensity. These results contribute to deciphering the critical biological principles underlying the regulatory mechanism of the in-cell Cu(I) concentration, and provide a basis for interpreting future studies that will take advantage of cysteine-to-serine mutations in Cu(I)-binding systems.

## 1. Introduction

Copper is an essential metal for cell function. Owing to its toxicity, cells possess a sophisticated and meticulous regulatory system that is responsible for shuttling the copper ions to their appropriate targets, while extruding the excess amount from the cell [1,2,3]. The proteins involved in this regulatory system share cysteine-based motifs—a common motif is CXXC (where X can be any amino acid), which has a high affinity for Cu(I) binding, allowing concurrently its rapid release to the next CXXC-containing protein target along the copper transport route [4,5]. Resolving the copper cycle in eukaryotic and prokaryotic systems is of utmost importance for several reasons: (1) Disruption of copper homeostasis has been shown to lead to various neurological diseases (such as Alzheimer’s, Parkinson’s, and Prion’s disease) [6,7]; (2) the copper cycle is used in the shuttling of metal-based therapeutic drugs such as cisplatin chemotropic compound [8,9,10,11]; (3) copper has been used for thousands of years as an antibacterial agent [12,13]. Hence, gaining an in-depth understanding of how these systems work, both in eukaryotic and prokaryotic cells, can assist in devising innovative strategies to control the in-cell Cu(I) concentration according to health needs, either by developing novel drug candidates or by designing a new generation of antibiotics. 

Many biophysical methods have been applied to characterize the proteins taking part in the copper cycle: (1) X-ray, nuclear magnetic resonance (NMR), and cryo-electron microscopy (EM) have been used to resolve the structures of various proteins [14,15,16,17,18,19,20]; (2) extended x-ray adsorption fine structure (EXAFS) was used to define the Cu(I) binding sites [21,22]; (3) single-molecule Forster resonance energy transfer (smFRET) has been employed to study the dynamics of these systems [23,24,25]; (4) electron paramagnetic resonance (EPR) measurements have been used to capture the various conformational states of these proteins [26,27,28,29]; (5) molecular simulations have been applied to fill many gaps of knowledge and to decipher unclear aspects of this intricate regulatory mechanism [30,31,32,33]. 

In order to compare a functional protein with a non-functional/inhibited/repressed protein, cysteine (Cys)-to-serine (Ser) mutations have often been applied in in vitro studies [17,34,35,36,37], relying on the hypothesis that the SXXS site is unable to bind Cu(I) ions. This hypothesis is mainly based on functional measurements, showing that double mutation of CXXC to SXXS aborts all functions of the hosting protein. For example, SXXS double mutation in the human Cox17 metallochaperone reduced cell growth [35], and a single serine mutation in the *Escherichia coli* CueR metal regulator (which has a different dithiolate double Cys motif) affected the transcription process [36]. Moreover, the Cys-to-Ser mutation in Cox17 and CueR led to a reduction in the stoichiometry of a number of bound Cu(I) ions. Nevertheless, EXAFS studies suggested that double Cys-to-Ser mutations of the human copper chaperone CCS can still bind Cu(I) ions, although in a distinct manner with respect to the wild-type (WT) protein, possibly leading to its oligomerization or precipitation (aggregation) [37]. An oligomerization of various ZuR monomers in the presence of Cys-to-Ser mutation was also observed in-cell [34]. Such oligomerization can be detected in vitro as aggregation or precipitation of the protein. Consistently, a theoretical study showed that Cu(I) binds to cysteine-based motif differently than to serine-based motif, but that Cu(I) binding may still occur in both cases [38]. 

Although it is clear that Cys-to-Ser mutations at the Cu(I) binding site markedly affect the function of these proteins, several open questions stand out: What is the mechanism? Are mutated Cu(I)-binding proteins still able to bind Cu(I) ions? Which rearrangements occur in these protein structures and how do they affect the proteins’ oligomerization state? Addressing these questions is essential for correctly interpreting future in vitro studies and for structural measurements, which aim at studying Cu(I) metabolism and homeostasis, by introducing these mutations to monitor specific aspects of the Cu(I) regulatory mechanism. 

Seeking to elucidate these unclear questions, the present study encompasses two proteins: The human ATP7B and the *E. coli* CueR metalloregulator, as prototypical examples of Cu(I)-binding proteins. ATP7B is located in the Golgi apparatus of the human cell, and is a part of a Cu(I) transport cycle including two other proteins: (1) The main copper transporter Ctr1, which transfers copper ions from the blood stream to the cytoplasmic domain; and (2) the metallochaperone Atox1, which transfers Cu(I) ions from Ctr1 to ATP7B. Atox1 interacts with the N-terminal domain of ATP7B, which contains six metal-binding domains (MBDs) interconnected by linkers. Each MBD (~7 kDa) displays a ferredoxin-like fold with a compact βαββαβ structure and a conserved metal-binding motif MXCXXC (X can be any amino acid), located in the solvent-exposed β1-α1 loop (also called the Cu loop), which binds Cu(I) ions. Atox1 is a small soluble cytosolic Cu(I) receptor composed of 68 amino acids and arranged in the same βαββαβ motif possessed by each MBD in ATP7B [39,40]. The similarity in structure between the chaperone and the target protein is the hallmark of the transporters taking part in copper homeostasis control.

Conversely, CueR is a metalloregulator protein that “senses” Cu(I) ions with very high affinity through a binding site that comprises two cysteine residues (C112 and C120). Cu(I) binding stimulates DNA recruitment and the transcription activation of two other metalloregulator proteins [25,29,41]. CueR exists as a homodimer; each monomer is composed of three domains: (1) A N-terminal DNA-binding domain, carrying two helix-turn-helix motifs; (2) a central dimerization domain, containing a 10-turn α-helix, which forms an antiparallel coiled-coil with the helix of the other monomer; and (3) a C-terminal metal-binding domain characterized by two conserved cysteine residues. This dithiolate motif contributes to the extremely high selectivity and sensitivity of Cu(I) ions, which reaches the 10^−21^ M range. In this manner, CueR can rapidly remove toxic copper ions from the cytoplasm.

In order to better understand how Cys-to-Ser mutations, localized in the binding site of Cu(I)-binding proteins, affect the proteins’ structure and aggregation, we carried out cross-linking experiments, circular dichroism (CD), EPR measurements, along with all-atom molecular dynamics (MD) simulations based on a classical (force field) and on a hybrid quantum-classical (QM/MM) description. As a result, we found that Cu(I) can still bind to its target proteins even in the presence of serine mutations; however, these change the aggregation state as well as the interaction of the hosting proteins with their biological partners. This study contributes to revealing the molecular basis for the impact of serine mutation on Cu(I) sensing and regulation. Hence, it provides precious information that will aid in guiding and interpreting future biophysical studies focused on deciphering the fine details of Cu(I) homeostasis control.

## 2. Results

### 2.1. The Effect of Serine Mutations on ATP7B MBD3-4 and Their Interaction with Atox1

Among the selected systems, we first inspected the structural and functional consequences of Cys-to-Ser mutations on two out of the six MBDs belonging to ATP7B. Namely, we studied MBD3 and MBD4, which are connected by an unstructured linker (PDB ID 2ROP) [42]. We expressed and purified WT-MBD3-4 (hereafter termed MBD3-4) and its mutant form, where the metal binding cysteine residues in each MBD (i.e., C268 and C271 in MBD3, and C370 and C373 in MBD4) were replaced by serines (hereafter termed SER-MBD3-4). We also expressed and purified WT-Atox1 metallochaperone, and then performed a series of experiments and simulations to explore the effect of Ser-to-Cys mutations on MBD3-4’s structure and aggregation, on the Cu(I) coordination sphere, and on the geometry of the Cu(I) binding site, as well as on its interaction with Atox1.

**Figure 1:**
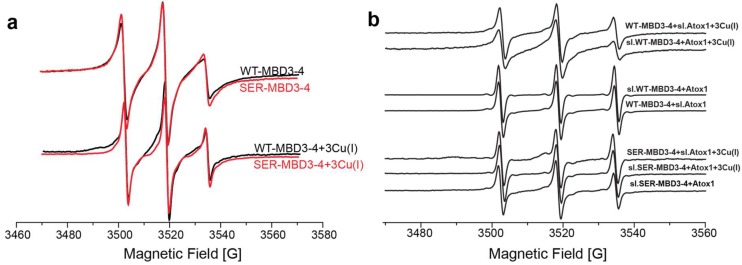
(**a**) Continuous wave electron paramagnetic resonance (CW-EPR) spectra of wild-type (WT)- metal-binding domains (MBD)3-4 (black line) and MBD3-4_C268S_C271S_C370S_C373S mutant where the metal binding cysteine residues in each MBD were replaced by serines (SER-MBD3-4; red line) in the presence and absence of Cu(I); (**b**) CW-EPR spectra of WT-MBD3-4 and SER-MBD3-4 in the presence and absence of Cu(I) and Atox1/sl-Atox1.

**Scheme A1:**
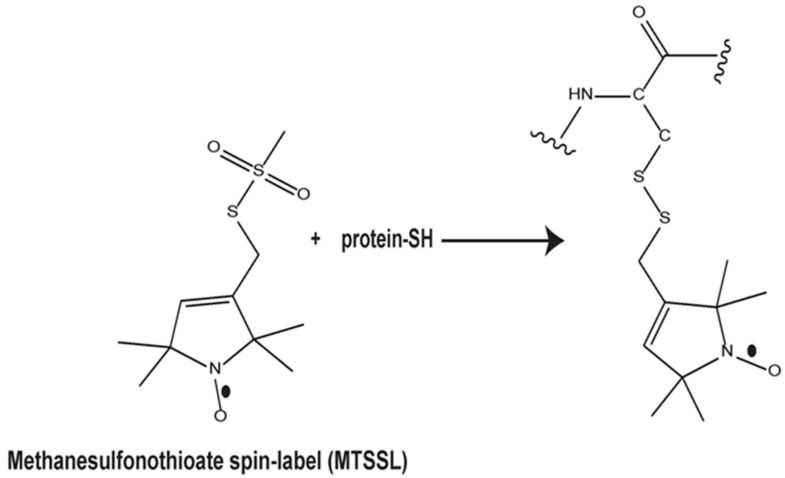
Spin-labeling reaction with MTSSL.

EPR spectroscopy has emerged as an excellent tool for addressing the unclear aspects of Cu(I) transport cycle, since it does not require crystallization and does not depend on the protein size. EPR measurement can be performed in buffer solution, and even weak interactions between proteins can be detected [43]. EPR’s strength lies in its sensitivity to both atomic level changes and nanoscale fluctuations. Continuous wave (CW) EPR can derive the dynamics of protein chains. The combination of CW-EPR with site-directed spin labeling has become widely used in biophysical research [44,45,46,47,48], where an electron spin introduced into diamagnetic proteins provides information on their local environment, and on the mobility of the protein domain [45]. The spin label, commonly attached to cysteine residues, is the methanesulfonothioate (MTSSL) nitroxide radical (see Scheme A1). Among the seven cysteine residues present in MBD3-4, four of them form the Cu(I) binding sites and could not be spin labeled in the presence of Cu(I) ions. Hence, spin labeling in WT MBD3-4 was limited to C305 (located in the loop between β3 and α2 of MBD3), C358, and C431 (located in β1 and β4 of MBD4, respectively). Figure 1a shows the CW-EPR spectra of MBD3-4 and SER-MBD3-4 (including the C268S, C271S, C370S, and C373S mutations).

The CW-EPR spectra exhibited only minor changes upon Ser mutations in MBD3-4, showing a bit of an increase in the dynamics and suggesting that the overall conformation of MBD3-4 did not change after their substitution. When Cu(I) was added to MBD3-4, a change in the CW-EPR spectrum was detected, suggesting that the spin-labels sites are a bit more dynamic upon Cu(I) binding [49]. Interestingly, the CW-EPR spectra in the presence of Cu(I) was similar when comparing MBD3-4 and SER-MBD3-4, pinpointing that Cu(I) binding can occur even in the presence of serines. However, when Atox1 was added to the solution (Figure 1b), different spectra emerged, indicating the higher mobility and dynamics of SER-MBD3-4 compared with its WT counterpart. We also spin-labeled Atox1 (sl-Atox1) at position C41 with MTSSL, and measured the CW-EPR spectra in the presence of MBD3-4 and SER-MBD3-4. Also here, the CW-EPR spectra of the two systems were different, suggesting that interactions between Atox1 and MBD3-4 in the presence of Ser mutations are not alike. These spectra suggest that SER-MBD3-4 undergoes some structural changes in the presence of Atox1, confirming that the CXXC motif is tremendously important for establishing appropriate interactions between Cu(I) transporters, and even apparently unharmful mutations may markedly affect their functional properties.

**Figure 2:**
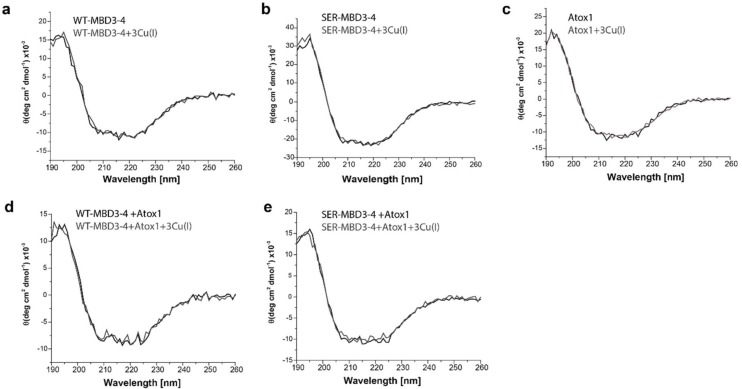
Circular dichroism (CD) spectra of (**a**) WT-MBD3-4; (**b**) SER-MBD3-4; (**c**) Atox1; (**d**) WT-MBD3-4 with Atox1; and (**e**) SER-MBD3-4 with Atox1 in the absence (black line) and presence of Cu(I) (gray line).

In order to determine whether the secondary structure of MBD3-4 is affected by the Ser mutations, we also performed CD measurements on MBD3-4 and SER-MBD3-4 in the absence and presence of Cu(I) and Atox1. The resulting CD spectra reveal that the secondary structure is preserved, and is affected neither by Ser mutations nor by Atox1 interactions (Figure 2). 

**Figure 3:**
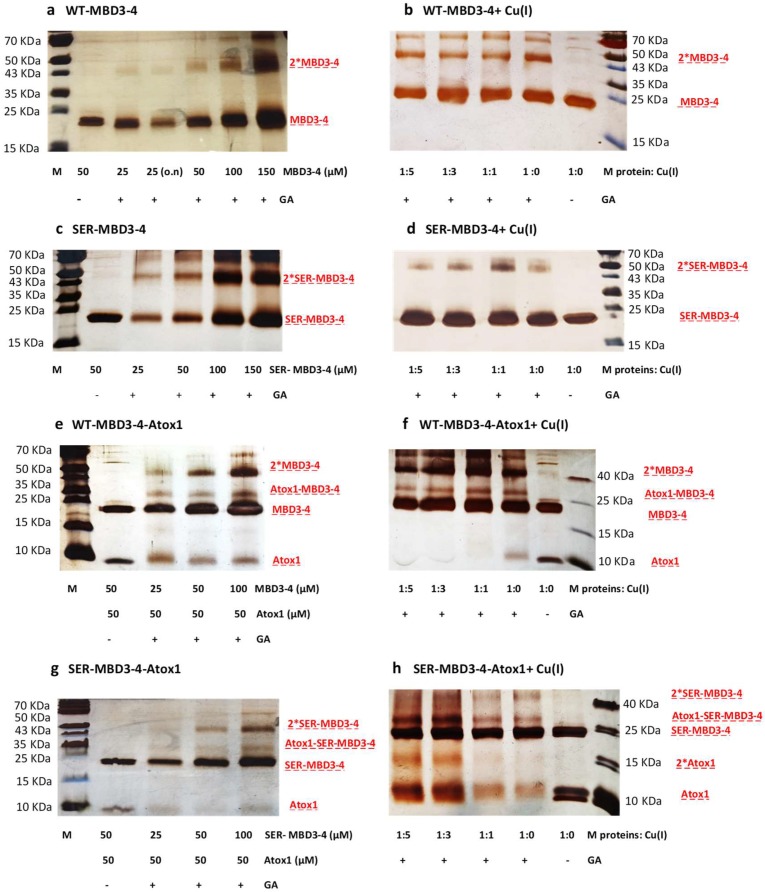
SDS-PAGE of cross-linking experiments of ATP7B (MBD3-4). (**a**) Increasing concentrations of WT-MBD3-4 were incubated 30 min or overnight (o.n.) with 0.1% glutaraldehyde (GA) at room temperature (RT); (**b**) WT-MBD3-4 (50 µM) was incubated for 30 min with Cu(I) at different ratios and then for 30 min with 0.1% GA at RT; (**c**) Same as (**a**) with SER-MBD3-4 mutant; (**d**) Same as (**b**) with SER-MBD3-4 mutant; (**e**) Atox1 (50 µM) was incubated for 30 min with increasing concentrations of WT-MBD3-4 and then for 30 min or overnight (o.n.) with 0.1% GA at RT; (**f**) WT-MBD3-4 and Atox1 (50 µM) were incubated for 30 min with Cu(I) at different ratios and then for 30 min with 0.1% GA at RT; (**g**) Same as (**e**) with SER-MBD3-4 mutant; (**h**) Same as (**f**) with SER-MBD3-4 mutant.

Next, we performed cross-linking experiments using glutaraldehyde (GA), which reacts with lysine residues. Figure 3, showing the SDS-PAGE run of MBD3-4 and SER-MBD3-4 as a function of the presence of Cu(I) and Atox1, reveals that: (1) Higher cross-linking between MBD3 and 4 occurs in Ser mutations and in the absence of Cu(I); (2) in the presence of Cu(I), the extent of SER-MBD3-4 cross-linking is reduced; (3) in the presence of Atox1, the extent of MBD3-4-Atox1 cross-linking is lower when introducing Ser mutations in MBD3-4, independently of the presence of Cu(I); and (4) in the presence of Atox1, the extent of cross-linking between SER-MBD3-4 units is higher. It is important to mention that each cross-linking experiment was repeated at least 4–5 times, to check for reproducibility. The cross-linking agrees well with the CW-EPR experiments. We can infer that in the presence of Ser mutations Cu(I) can still bind to MBD3-4; however, these mutations affect the aggregation of MBD3-4 as well as its interaction with its Atox1 partner. 

In order to characterize at the atomic level of detail the structural rearrangements occurring upon Cys-to-Ser mutations, we performed force field-based and QM/MM MD simulations [50]. At first, we inspected and compared the effect of metal binding on each MBD taken singularly (MBD3/4). Indeed, the NMR structure of MBD3-4 [42] (PDB ID 2ROP), the linker between the two domains, is not solved and the two domains do not interact. Consequently, even considering both domains in the simulation after building the linker between them would not add any relevant information. Therefore, we considered the single MBD3/4 and SER-MBD3/4, and we compared the metal free (apo) and the metal-bound (holo) states of MBD3 and MBD4.

**Figure 4:**
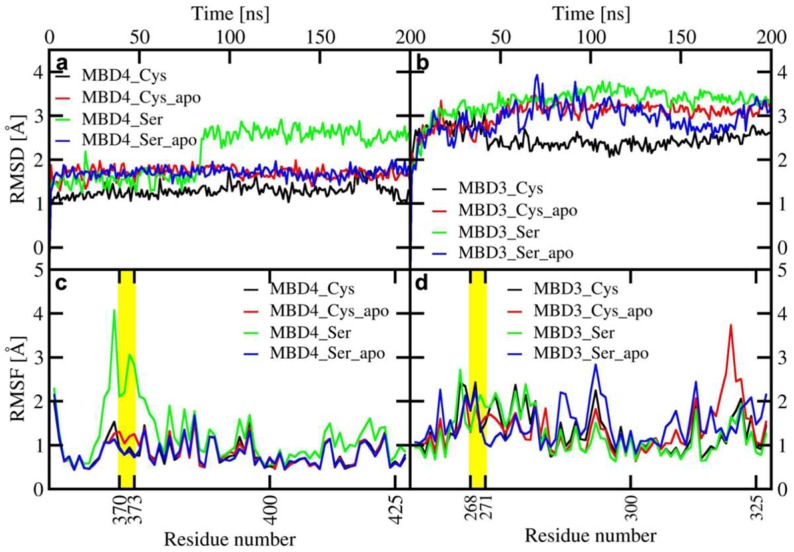
Upper panel: Root Mean Square Displacement (RMSD) of WT/SER-MBD4 vs. simulation time (ns) (**a**), and WT/SER-MBD3 (**b**). Lower panel: Root Mean Square Fluctuation (RMSF) plot of WT/SER-MBD4 (**c**), and WT/SER-MBD (**d**) calculated from the last 100 ns of cMD simulations. Black—holo WT-MBD3/4; red—apo WT-MBD3/4; green—holo SER-MBD3/4; blue—apo SER-MBD3/4). Cu(I)-binding regions are shown in yellow.

**Figure A1:**
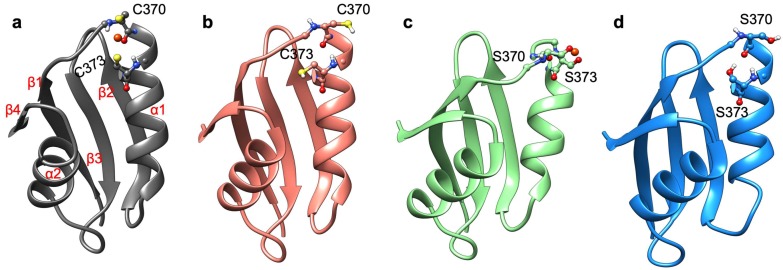
Most representativestrcutures of Cu(I)-bound and apo wild type (WT) and mutated (cysterine to serines) SER-MBD4. (**a**) WT-MBD4. Secondary structure motifs α1, α2 and β1–4 are labelled. (**b**) Apo WT-MBD4 (**c**) SER-MBD4. (**d**) Apo SER-MBD4, as extracted from a cluster analysis of the classical Molecular Dynamics (cMD) trajectories. Cu(I) ion is shown as orange sphere, sulfur atoms are colored in yellow and hydrogen atoms in white.

All MBD4 monomers, except the holo SER-MBD4, reached structural stability within a few ns of classical MD (cMD) simulations. Of these, MBD4 exhibited the lowest deviation from the initial structure in holo form, while apo forms of MBD4 and SER-MBD4 showed slightly higher deviation from their initial structure, as compared to holo MBD4. Instead, in the simulation of holo SER-MBD4 the deviation from its initial structure was the highest (Figure 4a). Additionally, Cu(I) binding to WT-MBD4 reduced the flexibility of the Cu loop (Figure 4). The flexibility of apo SER-MBD4 was almost identical to that of MBD4. However, the holo form was characterized by a significant overall increase of flexibility, which was particularly evident in the Cu loop (Figure 4). In line with these findings, the most representative structures of both apo and holo MBD4 and apo SER-MBD4 were very similar, whereas that of holo SER-MBD4 underwent a change in the Cu loop’s conformation and the unfolding of helix α1 (Figure A1).

**Figure A2:**
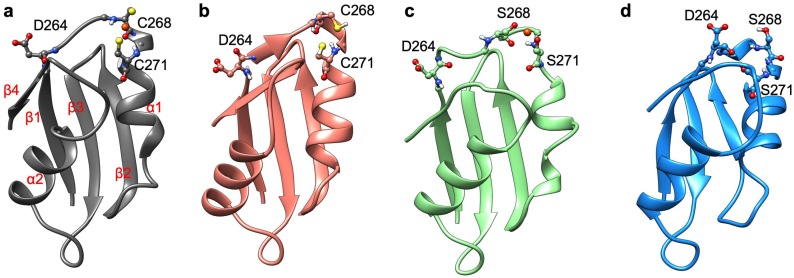
Most representativestrcutures of Cu(I)-bound and apo WT and SER-MBD3. (**a**) WT-MBD3. Secondary structure motifs α1, α2 and β1–4 are labelled. (**b**) Apo WT-MBD3 (**c**) SER-MBD3. (**d**) Apo SER-MBD3, as extracted from a cluster analysis of the cMD trajectories. Cu(I) ion is shown as orange sphere, sulfur atoms are colored in yellow and hydrogen atoms in white.

In contrast to MBD4, MBD3 monomers reached structural stability after longer simulation times (within a few tens of ns) and all of them were characterized by larger deviations from initial structures (Figure 4). Analogously to holo MBD4, holo MBD3’s structure became more rigid in the Cu(I)-binding region upon metal binding (Figure 4). Conversely, the metal coordination to SER-MBD3 stiffened most of the structure, while augmenting the flexibility of the Cu(I)-binding region (Figure 4). In spite of the similarity between the secondary structure of apo MBD3 and MBD4 (Figure A1b and Figure A2b) upon Cu(I) binding, α1 helix of MBD3 (Figure A2a) underwent partial unfolding. Interestingly, apo SER-MBD3 displayed a structure distinct from that of the corresponding apo SER-MBD4 form, due to the formation of an intramolecular hydrogen (H)-bond between Ser268 and Asp264, which, in all other MBD3 structures, was instead far apart (Figure A2d). Cu(I) binding caused a break of this H-bond, resulting in an unfolding of helix α1 (Figure A2c).

Next, cMD simulations were carried out on Atox1 in complex with apo MBD3 or MBD4 in order to inspect the structural stability and dynamical properties of the resulting adducts, and to assess their possible impact on Cu(I) transport. The adducts were built on the basis of Atox1 homodimer X-ray structure (PDB ID 1FEE) [4] by superimposing each MBD to one monomer of Atox1 as in our previous study [49]. Here, we considered a face-to-face model [49], of the complex accounting for three different possible states: The apo state, in which Cu(I) is not present in the system; and two holo states, with Cu(I) always bound to Atox1’s binding site. The two holo states were chosen since our goal was to estimate the tendency of formation for the initial encounter complex between the two proteins, along the Cu(I) transport route from Atox1 to ATP7B. In this latter mode, whereas the metal binding cysteines/serines of Atox1 are always considered to be deprotonated, those of MBD3/4 are either protonated or deprotonated.

**Figure 5:**
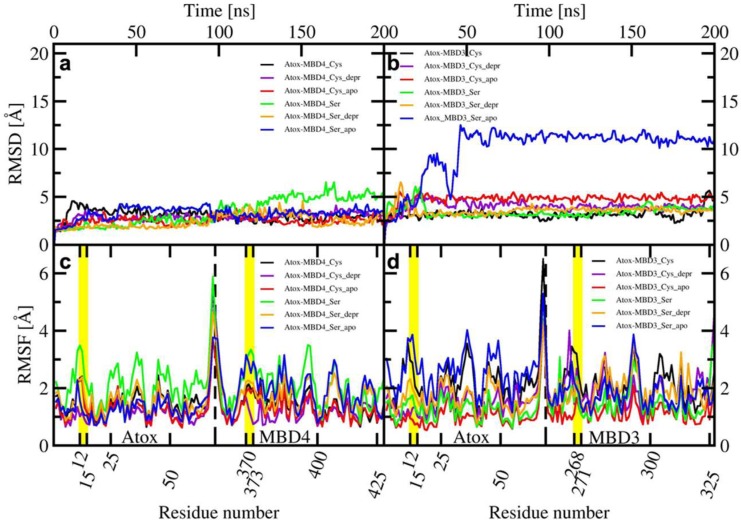
Upper Panel: Root Mean Square Displacement (RMSD) plot vs. simulation time (ns) of Atox1-WT/SER-MBD4 (**a**), and Atox1-WT/SER-MBD3 (**b**). Lower panel: Root Mean Square Fluctuation (RMSF) plot vs. the residue number of Atox1-WT/SER-MBD4 (**c**), and Atox1-WT/SER-MBD (**d**) calculated over the last 150 ns of MD trajectory. Black—holo Atox1-WT-MBD3/4; violet—holo Atox1-WT-MBD3/4_depr; red—apo Atox1-WT-MBD3/4; green—holo Atox1-SER-MBD3/4; orange—holo Atox1-SER-MBD3/4_depr; blue—apo SER-MBD3/4). Cu(I)-binding regions are highlighted in yellow.

**Figure A3:**
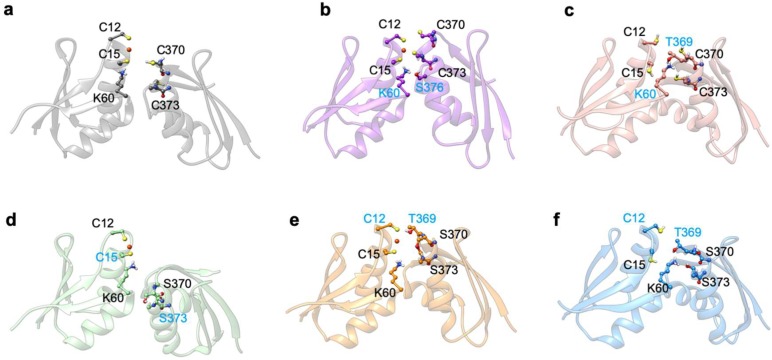
Structures of highest populated clusters of Atox1-MBD4 complexes. (**a**) Atox1-Cu(I)-MBD4. (**b**) Atox1-Cu(I)-MBD4-depr (**c**) Apo Atox1-MBD4. (**d**) Atox1-Cu(I)-SER-MBD4. (**e**) Atox1-Cu(I)-SER-MBD4-depr (**f**) Apo Atox1-SER-MBD4. Cu(I) is shown as orange sphere, sulfur atoms are colored in yellow and hydrogen atoms in white. Labels of residues that form inter-protein H-bonds are coloured in light blue (see Table A1, Table A2, Table A3 and Table A4 for details).

**Figure A4:**
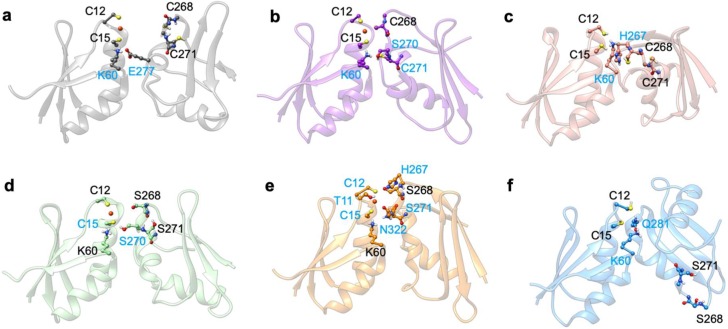
Structures of highest populated clusters of Atox1-MBD3 complexes. (**a**) Atox1-Cu(I)-MBD3. (**b**) Atox1-Cu(I)-MBD3-depr (**c**) Apo Atox1-MBD3. (**d**) Atox1-Cu(I)-SER-MBD3. (**e**) Atox1-Cu(I)-SER-MBD3-depr (**f**) Apo Atox1-SER-MBD3. Cu(I) is shown as orange sphere, sulfur atoms are colored in yellow and hydrogen atoms in white. Labels of residues that form inter-protein H-bonds are coloured in light blue (see Table A1, Table A2, Table A3 and Table A4 for details).

Namely, in the apo Atox1-MBD3/4 adducts, the cysteines/serines, which form the Cu(I) binding sites of MBD3/4 and Atox1 (i.e., C12/15@Atox1, C268/271@MBD3, S268/271@MBD3, C370/373@MBD4, and S370/373@MBD4) were all protonated. As reported in our previous study [49], the apo complexes involving Atox1-MBD4 and Atox1-MBD3 were stable during our cMD simulations (Figure 5a). This was possibly due to a weak H-bond forming between K60@Atox1 and T369@MBD4 (Figure A3c and Table A1), whereas the Atox1-MBD3 structure significantly differed from that of Atox1-MBD4 due to H-bond engagement between K60@Atox1 and H267@MBD3 (Figure A4c and Table A2).

When inserting the serine mutations, although the structure of the highest populated cluster of apo Atox1-SER-MBD4 does not differ significantly from that of Atox1-MBD4, and the H-bond formed between K60@Atox1 and T369@MBD4 appears to be of similar strength (Table A3), the flexibility of the adduct increased (Figure 5 and Figure A3f). Conversely, the Atox1-SER-MBD3 structure differed from that of Atox1-MBD3 (Figure A4f) due to the formation of a weak H-bond between K60@Atox1 and Q281@MBD3 (Table A4).

When simulating the holo adducts, force field-based MD, relying on predefined empirical parameters, is not designed to directly simulate bond breaking and the formation of the metal’s coordination sphere, which may occur upon Cu(I) binding, along with its delivery from Atox1 to MBD3/4 [51]. Therefore, in these simulations of the holo adducts, we needed to set a priori the Cu(I) coordination bonds, keeping them unchanged during the MD simulations, as in our previous study [49]. Here, we considered Cu(I) bound to Atox1, while C268/C271, S268/S271, C370/C373, and S370/S373 of MBD3 and MBD4, respectively, were kept protonated and no bond was set between Cu(I) and the cysteines of MBD3/4.

**Figure 6:**
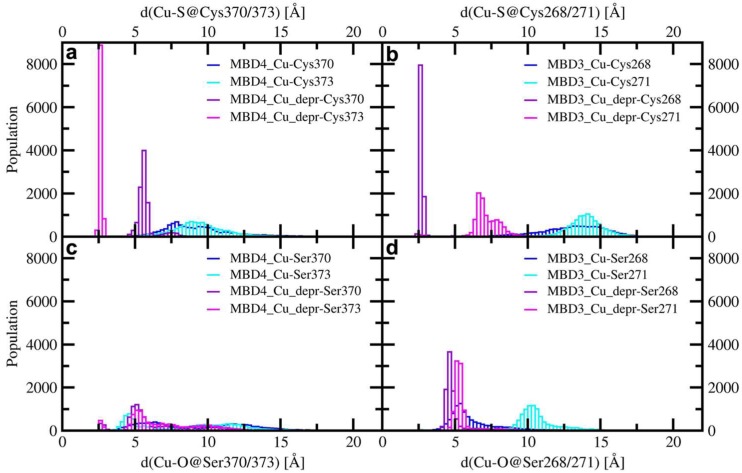
Distribution of distances between Cu(I) and the cysteine residues of MBD4 (**a**), and MBD3 (**b**), and the serine residues of MBD4 (**c**), and MBD3 (**d**) in the complexes of holo Atox1 with WT/SER-MBD3/4 considering for (**c**) and (**d**) the protonated and deprotonated cysteine residues (blue—the distance between Cu(I) and protonated S/O@Cys/Ser370/268; cyan—the distance between Cu(I) and protonated S/O@Cys/Ser373/271; violet—the distance between Cu(I) and deprotonated S/O@Cys/Ser370/268; magenta—the distance between Cu(I) and deprotonated S/O@Cys/Ser373/271).

This was done to study the short-lived encounter complex, which is supposed to form at the early stage of the excretion path [52]. As a result, Cu(I) binding to Atox1 affected the structural flexibility of the Atox1-Cu(I)-MBD4 heterodimer (Figure 5c). Although the most representative structure extracted from the MD trajectory appeared suitable for Cu(I) trafficking, the long distance between Cu(I), coordinated to Atox1, and C370/C373 of MBD4, would probably hamper Cu(I) transfer from Atox1 to these latter residues of MBD4 (Figure 6a and Figure A3a) [49]. In the Atox1-Cu(I)-MBD3 we again observed a partial unfolding of helix α1, (Figure A4a) and an increase in the distance between Cu(I) and C268/C271, compared with MBD4 (Figure 6b) [49]. 

In the presence of mutated Cu(I)-binding residues in Atox1-Cu(I)-SER-MBD4, the H-bond network was rearranged, with T369 pointing towards the Cu(I)-binding site of Atox1, whereas S370 and S373 point in different directions (Figure A3d). This is accompanied by a higher flexibility of the complex, compared with that of Atox1-Cu(I)-MBD4 (Figure 5c). This picture was reversed, however, when considering SER-MBD3, since the Atox1 and the MBD3 metal binding sites were closer to each other than in the WT counterpart (Figure 6c,d and Figure A4a,d) and the mutations contributed to stiffening the resulting Atox1-Cu(I)-SER-MBD3 adduct (Figure 5d). Among all holo systems detailed above, inter-protein H-bonds were formed between K60@Atox1 and E277@MBD3 in Atox1-Cu(I)-MBD3 (Table A2), and C15@Atox1 and S271@SER-MBD3 in Atox1-Cu(I)-SER-MBD3 (Table A4).

Cu(I) delivery from Atox1 to MBD3/4 required the formation of direct interactions between Cu(I) and the receiving cysteine/serine residues of the metal binding site. Hence, it was likely that during Cu(I) transport, C268/C271, S268/S71 of MBD3, and C370/C373, S370/S373 of MBD4 would either spontaneously deprotonate or their deprotonation will be induced by Cu(I) binding. Since cysteine’s side chain is more acidic than that of serine, deprotonation is expected to occur more easily in the WT proteins. In order to assess how the protonation of the Cys/Ser residues of the (SER)-MBD3/4’s Cu(I) binding motif could impact the stability of the Atox1-MBD3/4 adducts, we also considered the holo Atox1-(SER)-MBD3/4 complexes with deprotonated (depr) C(S)268/271@MBD3 and C(S)370/373@MBD4 residues. Since we wanted to investigate initial encounter complex between the two proteins, in these initial cMD simulations an explicit coordination bond between the Cu(I) and Cys/Ser residues of the metal binding site was absent. Hence, the interactions between Cu(I) and C(S)268/271@MBD3 or C(S)370/373@MBD4 were purely electrostatic [50]. 

As observed for the WT complex [49], the flexibility of the Atox1-Cu(I)-MBD4_depr adduct was lowered with respect to the other systems (Figure 5c). This was particularly evident in the Cu(I)-binding region of both proteins. The predominant structure obtained from the cMD trajectory displayed Cu(I) bound by two cysteine residues of Atox1 and strongly interacting with C373 from MBD4 (Figure 6a). The adduct was also stabilized by an H-bond between K60@Atox1 and S373@MBD4 (Table A1). Conversely, by introducing serine mutations, these residues did not directly interact with Cu(I) and the T369@MBD4 H-bonds did not interact with C12@Atox1 (Figure A4b,e and Table A3).

Deprotonation of C268 and C271 of MBD3 was previously found to increase the flexibility of the Atox1-Cu(I)-MBD3_depr complex (Figure 5), although Cu(I), bound by two Atox1 Cys residues, still strongly interacted with C268@MBD3 (Figure 5 and Figure A4a,b,d,e). Similarly to the WT system, deprotonation of S268 and S271 of SER-MBD3 also increased the flexibility of the Atox1-Cu(I)-SER-MBD3depr complex (Figure 5). The only difference between the WT and mutant adducts lay in the H-bond between H267@MBD3 and C12@Atox1 (Figure A3e and Figure A4e, Table A2 and Table A4). Interestingly, the distances between Cu(I) and S268/271@MBD3 were considerably shorter than those between Cu(I) and S370/373@MBD4 (Figure 6c,d).

Since an interaction between Cu(I) and MBD3/4-depr was established already during the cMD simulations, we also performed 5-ps long QM/MM MD simulations by considering in the QM region all possible Cu(I) binding residues as well as K60@Atox1. This allowed us to inspect more accurately the structure of the metal coordination sphere and its possible rearrangements upon the formation of the adducts. This was done on both holo adducts and considering WT and SER-MBDs. 

**Figure 7:**
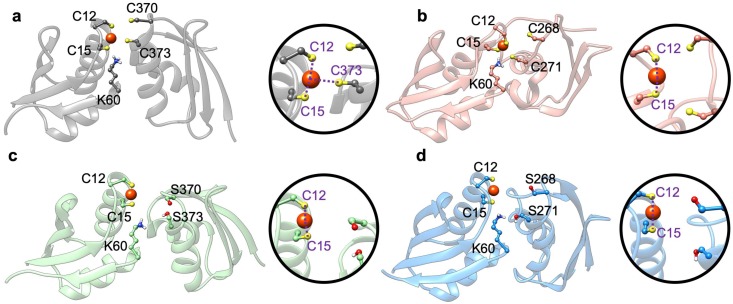
Structures of Atox1-WT-MBD4_depr (**a**), Atox1-WT-MBD3_depr (**b**), Atox1-SER-MBD4_depr (**c**), and Atox1-SER-MBD3_depr (**d**) resulting from 5 ps-long QM/MM MD simulations. Cu(I) is shown as an orange sphere, sulfur atoms are yellow, and hydrogen atoms are white. In spheres are close up views of Cu(I) ion and its coordination sphere of each system. Coordination bonds are shown as violet dashed lines. Only residues coordinating Cu(I) ion are named.

Since in the Atox1-Cu(I)-(SER)-MBD3/4 complex the cysteine/serine residues of MBD were far apart, the metal remained bi-coordinated to the two Atox1 cysteins belonging to the metal binding motif, and no interaction with MBD3/4 was established along the QM/MM MD trajectory. Conversely, in some simulations of the Atox1-Cu(I)-(SER)-MBD3/4-depr complexes the metal coordination sphere strikingly rearranged with respect to that of cMD trajectories. The metal coordination motif in Atox1-Cu(I)-MBD4_depr remains close to that obtained from cMD (i.e., Cu(I) was tri-coordinated, by C12 and C15 of Atox1, and C373 of MBD4), in Atox1-Cu(I)-MBD3_depr Cu(I) it became bi-coordinated by C12 and C15 of Atox1, whereas C271 and C268 of MBD3 moved far apart from the metal, suggesting that metal transfer is unlikely for MBD3, and that Cys-to Ser replacement markedly affects metal coordination. Strikingly, in the presence of deprotonated S268/271 in MBD3, and S370/373 in MBD4, K60 quickly protonated S271@MBD3 and S373@MBD4 consistently with the lower acidity of serine residues. As a result, both serine residues moved away from Cu(I), which remained coordinated only by Atox1 (Figure 7c,d). This made Cu(I) delivery to SER-MBD3/4 less likely. Although density functional theory-BLYP simulations may underestimate the free energy barrier for the proton transfer [53], our results, even though they are single-replica simulation, disclosed a tendency of serines to remain in their neutral state.

Consistent with these findings, K60 is well known as a critical residue for the interaction and stability of the complex [54], as well as for the transport mechanism of Cu(I) from Atox1 to MBD4. Moreover, we showed that the change in the protonation state of the metal-binding motif, known to affect transport [55], was most likely different in the presence of Cys-to-Ser mutations. This strikingly affected the structural, and possibly the functional, properties of the hosting proteins, which is consistent with the differences observed in CW-EPR spectra and the cross-linking experiments.

### 2.2. The Effect of Serine Mutations on CueR Protein

**Figure 8:**
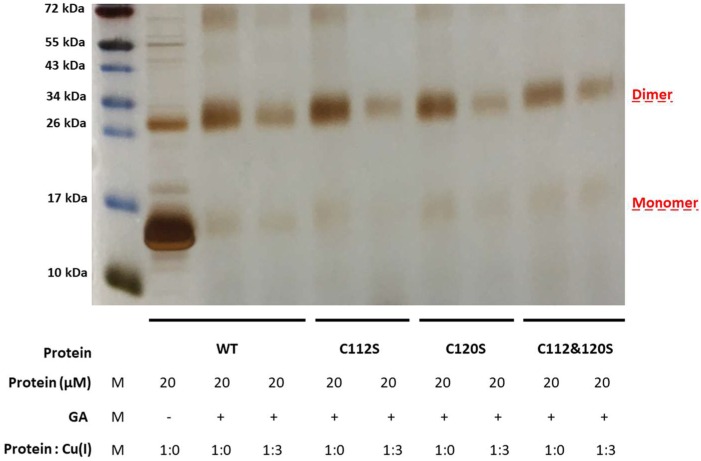
SDS-PAGE of cross-linking experiments of CueR. WT and mutated proteins (CueR_C112S, CueR_C120S, and CueR_C112S_C120S) were incubated with or without Cu(I) for 30 min at a 1:3 ratio and then for another 30 min with 0.6% GA at RT.

**Figure 9:**
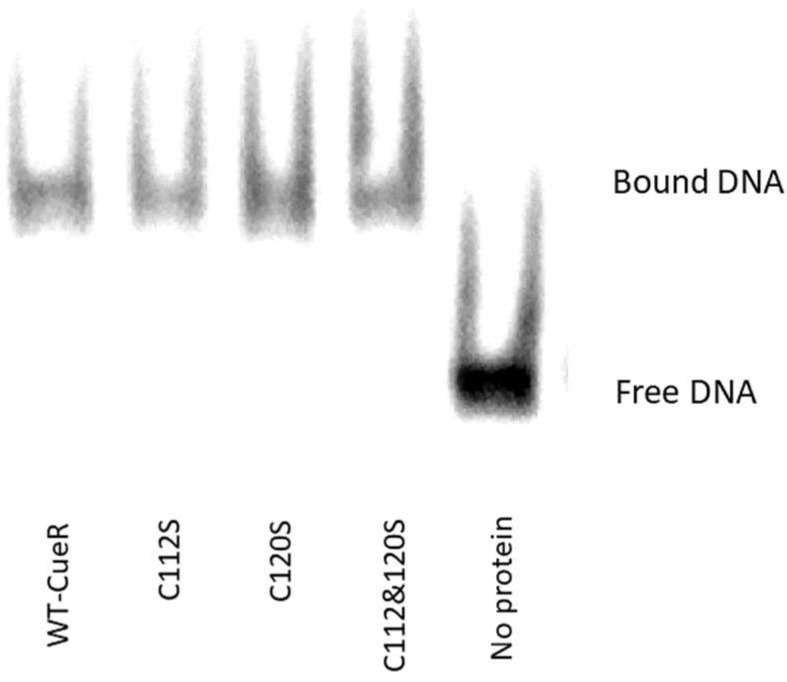
Electrophoresis mobility shift assay by fluorescence shows that the mutant CueR proteins bind the promoter similarly to their WT counterpart. Protein-Cy5_DNA complexes, 3 µM:1.5 µM, were formed in 40 µL incubation buffer. Protein-DNA complexes were resolved on 4.5% (37:1) native polyacrylamide gel and samples were imaged by Typhoon phosphorimager.

In order to verify that the observed modifications on the structure, as well as the aggregation propensity, were not system dependent, we also carried out cross-linking experiments on the CueR metalloregulator. This protein is found in *E. coli* as a homodimer which contains two Cu(I) binding sites within the C112 and C120 residues, located in a disordered loop of each monomer. To this end, we created three mutants of CueR: CueR_C112S, CueR_C120S, and CueR_C112S_C120S. The WT-CueR cross-linking SDS-PAGE (Figure 8) displays two bands: one corresponding to the CueR monomer, and the higher band to the CueR dimer. For WT-CueR, the SDS-PAGE indicated a reduction in the dimer form as a function of Cu(I) ion binding. Similar behavior was seen with the three mutants, where a larger reduction in the dimer’s band was observed. This implies that the addition of Cu(I) affected the protein folding in a manner that possibly induces protein aggregation (precipitation of various CueR diners). Moreover, the C112S and C120S mutations accelerated this aggregation (both the dimer and the monomer bands were reduced). In order to investigate the behavior of these mutations, we confirmed that they can bind DNA same as WT-CueR, by performing an electrophoresis mobility shift assay by fluorescence (Figure 9).

**Figure 10:**
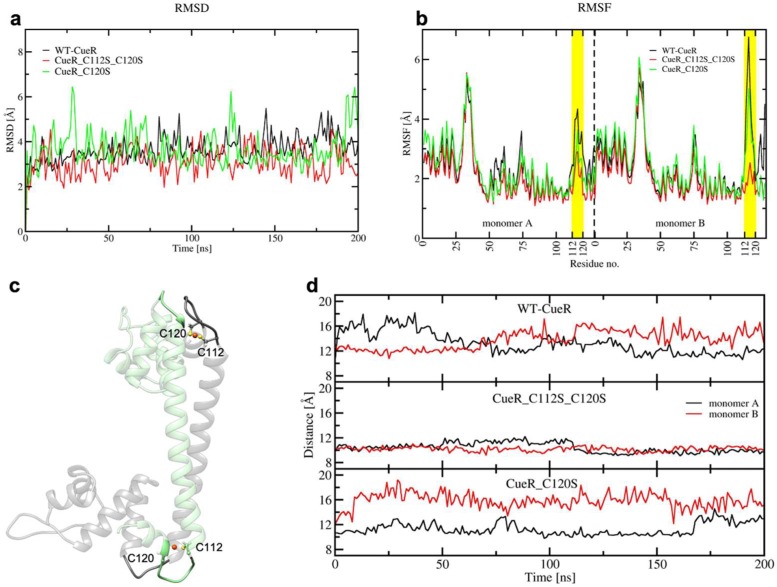
(**a**) Root Mean Square Deviation (RMSD) plot of WT_CueR (black), CueR_C112S_C120S (red), and CueR_C120S (green) during the 200 ns of classical MD (cMD) simulations. (**b**) Root Mean Square Fluctuation (RMSF) plot of WT_CueR (black), CueR_C112S_C120S (red), and CueR_C120S (green) during the 200 ns of classical MD simulations. Cu(I)-binding regions are denoted in yellow. (**c**) The initial structure of CueR, based on its crystal structure (PDB ID 1Q05). Separate monomers are denoted in green and black, respectively. C112, C120, and Cu are shown as balls and sticks. Loops (residues 72–76 and 112–120) are depicted in opaque new cartoons, whereas the rest of the protein is depicted as a transparent new cartoon. (**d**) Distances between the center of masses of two loops (residues 72–76 and 112–120) during the 200 ns cMD. WT_CueR—top, CueR_C112_C120S—middle, CueR_C120S—bottom. Monomers A and B are shown in black and red, respectively.

To characterize, at the atomic level, the impact of introducing serines, we also performed 200 ns-long cMD simulations of three systems (WT-CueR, CueR_C112S_C120S, and CueR_C120S). Simulations were performed without DNA, as this latter was absent in the crystal structure used to model this system. Here, we did not consider the CueR_C112S model as we did not expect any major difference in the behavior of CueR_112S with respect to CueR_C120S. RMSD and RMSF (Figure 10a,b) revealed that all systems are rather flexible. Nevertheless, the flexibility was mostly localized on the metal binding loop, being the highest in WT-CueR, whereas it decreased progressively in the CueR_C120S and CueR_C112S_C120S systems.

**Figure 11:**
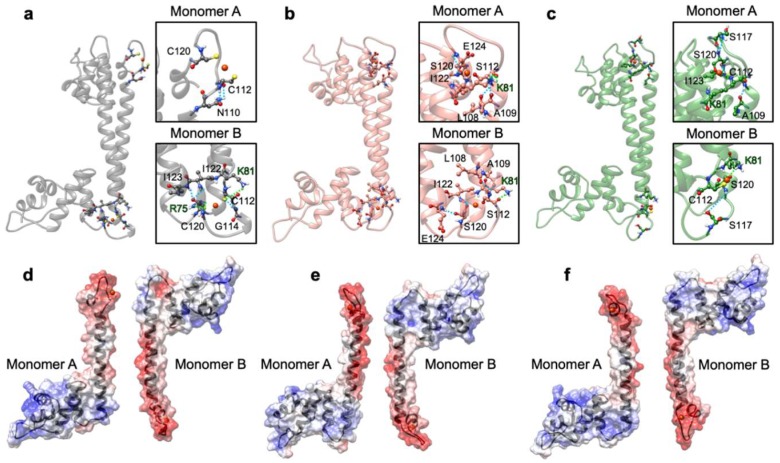
The most representative structure of the WT_CueR (**a**), CueR_C112S_C120S (**b**), and CueR_C120S (**c**) systems as extracted from a cluster analysis of classical MD trajectories. The corresponding Cu(I)-binding regions are reported in squares; Cu(I), its coordinating residues Cu(I), and the nearby H-bonded residues are depicted as balls and sticks. Nitrogen atoms are in blue, oxygen in red, sulphur in yellow, copper in orange, and hydrogen in white. Electrostatic potential surfaces are shown below (WT_CueR (**d**), CueR_C112S_C120S (**e**), and CueR_C120S (**f**)). The red and blue colors of the surface denote a negative and positive charge, respectively. Monomers were split and rotated by 90 degrees to show more clearly their interacting surfaces.

Next, we monitored the distance between the centers of mass of the Cu(I)-binding loop in one monomer (residues ranging from C112 to C120), and the loop interacting with it in the other monomer (residues from 72 to 76), to assess how the distinct flexibility of the WT and mutant CueR affects the aggregation state of the protein (Figure 10c,d). This analysis strikingly revealed that the distance between the two loops was smallest in the CueR_C112S_C120S system, in line with the increased stiffness of the homodimer and its larger aggregation propensity, experimentally observed. Interestingly, in the WT crystal structure, C112 and C120, and Cu(I) located between them (termed the Cu-bridge hereafter) was turned inside the dimer (Figure 10c), whereas during the cMD simulations in WT-CueR and CueR_C120S the Cu-bridge changed its conformation in one monomer, becoming exposed to solvent, as opposed to the simulation of CueR_C112S_C120S in which both Cu-bridges remained in their initial conformation. This was most likely due to the shorter distance of the O-Cu bond with respect to the S-Cu one, which made the Cu(I)-bridge more rigid and less prone to undergo conformational rearrangements. We believe that the conformational change was due to the induced mutation, however we cannot completely rule out the possibility that it could be also observed in CueR_C112S_C120S system if we would sample the system for longer time and/or if we would consider more than one replica. Figure 11 depicts a representative structure from the highest populated clusters of each system, along with a close-up view of the Cu(I)-binding loops and H-bonds formed by the residues coordinating the Cu(I) ion. The panels in Figure 11d–f, reporting the electrostatic surfaces of each separate monomer, revealed that the Cu-bridge region in the CueR_C112S_C120S system was slightly more electro-negative compared with the other two systems. This rationalizes the higher binding also observed from an electrostatic perspective. 

As the classical force fields have well known deficiencies when treating metal ions, we finally carried out QM/MM MD simulations on the most representative structures extracted from a cluster analysis of cMD trajectories for each system in order to refine the metal coordination sphere (Table A5). These simulations did not reveal any significant rearrangement in the metal geometry or in the interaction mode of the metal binding site with its facing monomer. Hence, also from QM/MM MD simulation we confirmed that also for CueR, Cys-to-Ser mutations influenced the oligomerization state of the protein. This may lead to altered protein functionality. 

## 3. Discussion

Copper is a vital transition metal ion used for biological processes and cell functions. Most Cu(I) transporters or Cu(I)-sensing proteins share conserved metallothionine motifs, which are well suited for feeling and binding Cu(I), even when present at extremely low concentrations in the cytosol [16,20]. Cysteines are characterized by a distinctive affinity for Cu(I) ions, making them a uniquely appropriate amino acid for maintaining Cu(I) homeostasis [56,57]. Cys-to-Ser mutation is often employed in Cu(I) binding proteins to inspect selected mechanistic aspects [17,34,36], based on the similarity between these two amino acids. This substitution relies on the hypothesis that, in spite of the similarity between cysteines and serines, the SXXS motif will not be able to bind to the Cu(I) ion, and therefore will form non-functional proteins. This proposal is mainly based on functional measurements, which showed that double mutation of CXXC to SXXS aborts all functions [34,36,37]. However, in vitro studies to validate this hypothesis and to clarify the specific variations occurring at both the structural and functional levels have not hitherto been performed. 

Here, we faced this unresolved aspect by studying the impact of Cys-to-Ser mutations in two prototypical Cu(I)-binding proteins, both sharing a CXXC Cu(I) binding site. Namely, we focused on CueR protein, which can sense the presence of Cu(I) in the cytosol at zeptomolar concentrations, and on two MBDs of ATP7B protein, critically involved in the Cu(I) excretion route from the Golgi apparatus. To this end, we employed a combination of experimental (EPR, CD, cross linking experiments) and computational (classical and quantum-classical MD simulations) techniques.

As a result, the CD experiments showed that the secondary structure of these proteins was not affected by Cys-to-Ser mutations. In addition, for CueR these mutations did not interfere with the protein-DNA interactions and the DNA binding propensity. However, our study unambiguously revealed that replacing Ser with Cys affects the oligomerization state of the hosting protein, as shown by the CW-EPR and cross-linking experiments for the CueR, ATP7B-MBD34 systems. Importantly, both the experimental and the simulation data consistently showed that Cu(I) can still bind to proteins after the Cys-to-Ser mutations. In addition, molecular simulations revealed that along the metal transport route mediated by Atox1-ATP7B the serine residues will most likely remain protonated, or if deprotonated, they would most likely easily sequester a proton from K60, a critically important residue for adduct stability, as indicated by mutagenesis studies [55]. This would either hamper the metal’s release from Atox1 to the target MBD4 protein or affect the stability of the Atox1-ATP7B adduct. Conversely, molecular simulations also revealed that in the CueR homodimer, the Cys-to-Ser replacement affected the stability of the homodimer, making it stiffer due to greater electrostatic complementarity and the engagement of H-bonds between monomers. Hence, the lower acidity of serine residues, its higher electronegativity, as well as their larger H-bonding power appear to be critical elements for maintaining the stability of the Cu(I)-binding proteins. This altered stability seemed to result in increased aggregation and oligomerization of the mutated proteins in vitro, and it possibly affected the interaction between the two proteins throughout the copper transport cycle.

Our outcomes unprecedentedly contribute to elucidating the fundamental principles underlying the perfect complementarity between Cu(I) and cysteine amino acids, and show that even a simple change of S to the O atom may strongly and adversely affect the function of Cu(I)-binding proteins. This information may be of importance in interpreting future biophysical studies taking advantage of this type of mutation. This may also foster the discovery of novel Cu(I)-binding small molecules or peptides, able to restore/modulate Cu(I) homeostasis (de)regulation, possibly paving the way to novel therapeutic strategies able to tackle human diseases deriving from Cu(I)-dyshomeostasis.

## 4. Materials and Methods

### 4.1. Cloning, Expression, and Purification of ATP7B 3-4 Metal Binding Domains

The ATP7B 3-4 metal binding domains’ (MBD3-4) gene was first amplified by PCR using primers containing specific ATP7B MBD3-4 sequences and flanking regions that corresponded to the expression vector sequences pTYB12. Forward primer of ATP7B MBD3-4: 5′-GTTGTACAGAATGCTGGTCATATGAGACCTTTATCTTCTGCTAAC-3′. Reverse primer of ATP7B MBD3-4: 5′-GTCACCCGGGCTCGAGGAATTTCAGTGGTTTCCAAGAGGGTTAGT-3′. This amplicon was cloned to pTYB12 vector by restriction-free cloning [58]. pTYB12 is a cloning and expression vector that allows the overexpression of the ATP7B MBD3-4 as a fusion to a self-cleavable intein tag. The self-cleavage activity of the intein allows the release of ATP7B MBD3-4 from the chitin-bound intein tag. The clone was expressed in *E. coli* strain Origami 2. The starter from stock glycerol was grown at 37 °C to an optical density of 0.5–0.6 (OD_600_) using terrific broth (TB) medium supplemented with ampicillin and tetracycline as selection factors, then induced with 1 mM isopropyl-β-d-thiogalactopyranoside (IPTG) at 18 °C overnight. Bacteria was then harvested by centrifugation at 10,000 rpm for 30 min. Then, the pellet was re-suspended in lysis buffer (25 mM Na_2_HPO_4_, 150 mM NaCl, 20 mM PMSF, 1% triton, pH 8.8) and sonicated (10 min of pulse 30 s of 40% amplitude). Finally, lysate was centrifuged at 14,500 rpm for 30 min and the supernatant was kept.

In order to purify the native ATP7B MBD3-4, the lysate was loaded on the chitin bead column, allowing the ATP7B MBD3-4-intein to bind to the resin through its chitin-binding tag. Resin was next washed with 50-column volumes of lysis buffer. Then, 5 mL dithiothreitol (50 mM DTT) was added and incubated for 48 h at 4 °C to perform self-cleavage of the intein. Elution fractions were then collected from the column using the chitin column buffer (pH = 8.8) and checked by 14% Tricine SDS-PAGE.

Atox1 expression and purification is similar to the expression of ATP7B and was described in a series of publications [26,27,59].

### 4.2. CueR Cloning and Expression Protocol

CueR was isolated by PCR using *E. coli* genomic DNA with primers specific to the CueR N-terminal (5′-GCAGCGGCCTGGTGCCGCGCGGCAGCATGAACATCAGCGATGTAGCAAAAATTACCGGCC-3′) and C-terminal (3′-GTCCACCAGTCATGCTAGCCATATGTCACCCTGCCCGATGATGACAGCAGC-5′). These primers also contain flanking sequences of the pET28a expression vector. The amplicon was cloned to the pET28a expression vector by the free ligation PCR technique. The same procedure was used to generate the different mutations, using specific primers containing the desired mutation. The CueR constructs were expressed in BL21 cells, which were grown to an optical density of 0.6 at 600 nm and induced with 1 mM isopropyl-β-d-thiogalactopyranoside (IPTG, CALBIOCHEM) overnight at 17 °C. The cells were then harvested by centrifugation at 10,000× *g* for 30 min. Pellets were resuspended in Tris buffer (25 mM Tris-base, 300 mM NaCl, pH 8.0), and 0.5% Triton X-100 was added to the cells’ lysis reagent, treated with homogenizer and passed through a Microfluidizer processor. Next, 2 mM of PMSF protease inhibitor (Sigma) were added to the lysed culture and centrifuged at 4 °C for 30 min at 20,000× *g*. The soluble fraction was separated from the pellet fraction and the pellet was suspended with Tris buffer at a 1:10 (g:mL) ratio according to the amount of growth. The protein was then purified from a soluble fraction by Ni-NTA agarose beads (Thermo Fisher Scientific), according to the manufacturer’s protocol using Tris buffer with 10 mM imidazole as the elution buffer.

### 4.3. Spin Labeling

The spin-labeling process was performed in the presence of Cu(I) ions in order to prevent spin-labeling of cysteine residues involved in Cu(I) binding. Before labeling, 10 mM DTT was added to the protein solution and mixed overnight (o.n.) at 4 °C. DTT was dialyzed out using 1 kDa dialysis cassettes (Pierce). Next, 0.25 mg of *S*-(2,2,5,5-tetramethyl-2,5-dihydro-1*H*-pyrrol-3-yl)methyl methanesulfonothioate (MTSSL, TRC) was dissolved in 15 μL Dimethyl sulfoxide (DMSO, Bio lab) was added to 0.75 mL of 0.01 mM protein solution (20-fold molar excess of MTSSL). The protein solution was then vortexed overnight at 4 °C. The free spin label and Cu(I) ions were removed by several dialysis cycles over 4 days. The mass of the spin-labeled protein was confirmed by a mass spectrometer, and the concentration was determined by BCA assay. 

### 4.4. Cu(I) Addition

After spin-labeling and all the dialysis steps were carried out for removal of free spin labels from the solution, we found no Cu(I) ions in the protein solution, which was verified by CW-EPR experiments in the presence of 0.1 mM KCN.

For EPR measurements: Cu(I) (Tetrakis (acetonitrile) copper(I) hexafluorophosphate) was added to the protein solution under nitrogen gas to preserve the anaerobic conditions. No Cu(II) EPR signal was observed at any time. 

### 4.5. Cross-Linking Experiments

Cross-linking reactions were carried out with 0.1% Glutaraldehyde (GA) for ATP7B or 0.6% CueR protein at RT for 30 min in phosphate buffer (25 mM Na_2_HPO_4_, 150 mM NaCl pH = 8), to a 50 µL final volume. Protein concentrations were 50 μM for ATP7B or 20 μM for CueR. The reactions were stopped by Tris-HCl buffer pH = 7.4 in a final concentration of 0.5 mM. Cross-linking experiments with copper: The proteins were incubated for 30 min with Cu(l) at different ratios and then for 30 min with GA at RT.

### 4.6. X-Band CW EPR Experiments

CW-EPR spectra were recorded using an E500 Elexsys Bruker spectrometer operating at 9.0−9.5 GHz equipped with a super-high-sensitivity CW resonator. The spectra were recorded at room temperature (292 ± 5 K), at a microwave power of 20.0 mW, a modulation amplitude of 1.0 G, a time constant of 60 ms, and a receiver gain of 60.0 dB. The samples were measured in 1.0 mm quartz tubes (Wilmad-LabGlass, Vineland, NJ, USA). CW-EPR simulations were carried out using MATLAB, with the EasySpin toolbox [60]. The final spin-labeled protein concentration was between 0.01 and 0.03 mM.

### 4.7. Circular Dichroism Characterization 

Circular Dichroism (CD) measurements were performed using a Chirascan spectrometer (Applied Photophysics, UK) at room temperature. Measurements were carried out in a 1 cm optical path length cell. The data were recorded from 190–260 nm with a step size and a bandwidth of 1 nm. Spectra were obtained after background subtraction. Circular dichroism measurements were conducted on MBD3-4, MBD3-4(Ser), and Atox1 after dialysis steps were carried out with water.

### 4.8. Electrophoresis Mobility Shift Assay by Fluorescence 

The copA promoter DNA fragment (5′-/5Cy5/TTGACCTTCCCCTTGCTGGAAGGTTTA-3′) that binds CueR protein was labeled using cy5 fluorescent dye. Protein-DNA complexes, 3 µM:1.5 µM, were formed in 40 µL incubation buffer (25 mM pH 8 Tris-base, 250 mM NaCl, 5% *v*/*v* glycerol) for 30 min at RT, then formaldehyde crosslinking assays were performed. Formaldehyde was added to a final concentration of 0.74% and the reactions were incubated for a further 30 min in ice. The crosslinked protein-DNA complexes were resolved on 4.5% (37:1) native polyacrylamide gels, which were pre-run at 20 mA for 1 h. The samples were loaded onto gel running at 10 mA for an additional hour. The gels were imaged by Typhoon phosphorimager (Typhoon FLA 9500).

### 4.9. Modeling of Systems

First, we prepared WT and mutated MBD3 and MBD4 (SER-MBD3 and SER-MBD4), both in the apo and holo states. The models were built based on their NMR structure in the apo form (PDB ID 2ROP) [42]. We initially considered the apo adduct in which the cysteine residues that bind Cu(I) in Atox1 were considered in a protonated form, as well as the cysteins and their mutant serines in MBD3 and MBD4. The protonation states of other ionizable residues were monitored with propKa [61].

In order to build the Cu(I)-bound systems, we superimposed both MBD3/4 to one monomer of the Cu(I)-bound Atox1 homodimer X-ray structure (PDB ID 1FEE) [4]. Owing to the high structural similarity of MBD3/4 to Atox1 and the shared MXCXXC motif, we adopted the same position of Cu(I), observed in Atox1, also for MBD3/4. To mutate cysteine residues to serine ones, we simply changed sulphur atoms to oxygen atoms.

For the holo Atox1-Cu(I)-MBD3/4 adducts, we built four model systems for WT MBD3/4 and four for SER-MBD3/4, in which Cu(I)-bound Atox1 formed dimers with apo MBD3 and MBD4, considering their C(S)268/370 and C(S)271/373 residues either in the protonated or deprotonated form. Namely, the cysteine residues binding Cu(I) in Atox1 were considered in a deprotonated form, whereas the cysteines/serines on MBD3/4 were considered either in protonated or deprotonated forms. In these models no explicit coordination bond is considered between the Cu(I) and C(S)268/370 and C(S)271/373 either in protonated or deprotonated forms.

For CueR, we initially took the crystal structure (PDB ID 1Q05) [41] of a homodimer and modelled missing parts in one monomer by superimposing it on the second monomer where the missing residues were present, to build a model with the cysteine residues coordinating Cu(I) (C112-C120). Next, we mutated C120 in both monomers to S120 by changing sulphur atoms to oxygen atoms, obtaining the CueR_C120S system. Finally, we also mutated the second Cu(I)-binding cysteine residue (C112) to serine to build the CueR_C112S_C120S model.

### 4.10. Classical Molecular Dynamics

All models were relaxed by performing classical MD simulations using an Amber parm14SB-ILDN force field for treating the protein [62]. For the Cu(I) and Cys/Ser residues coordinating it, we used parameters from Op’t Holt and Merz [63]. After performing QM/MM MD simulations, we adapted the bond distances and angles to the average obtained from these simulations, while maintaining the force constants unvaried. The same bonded model was used for MBD3/4 and CueR systems. All systems were solvated in explicit water using the TIP3P model [64] and Na^+^ ions were added to reach the neutral charge of the system. 

We used the Amber18 code [65] cuda program. After a careful equilibration, based on the geometry optimization and gentle heating in the NVT ensemble, 200 ns of cMD NPT simulations were carried out using the Berendsen barostat and Langevin thermostat [66,67]. Particle Mesh Ewald has been used to treat long-range electrostatics, and the time step in simulations was 2 fs.

The root mean square displacement (RMSD) and fluctuations (RMSF) were calculated with the *cpptraj* tool of Ambertools18 on the cMD trajectory. The same software was used for cluster analysis using a hierarchical agglomerate approach, which was used on all systems, as described before (on frames ranging from 20 to 200 ns from simulations for all systems) [65]. The cutoff for clustering was set to 1.5 Å for all monomeric MBD4 systems, 2.0 Å for all monomeric MBD3 systems, 2.5 Å for all Atox1-MBD3/4 complexes, and 4.0 Å for CueR systems. The electrostatic potential surface of each system was calculated based on the corresponding structure of the highest populated cluster using the PDB2PQR webserver [68]. Figures were done with Chimera1.12 software [69]. 

### 4.11. QM/MM Simulations

In order to relax Cu(I) coordination sphere, the most representative frames extracted from the MD trajectory were used as starting structures for QM/MM (i.e., quantum mechanics (Born Oppenheimer)/molecular mechanics) MD simulations, using the CP2K code [70,71]. This method treats part of the system at the QM level, usually the metal binding portion of the system, whereas the remaining part of the protein, the solvent, and the counter ions are treated at the MM level [51].

In these simulations, we considered the QM region Cu(I) and the side chains of residues coordinating it (C/S268 and C/S271 in (SER)-MBD3, C/S370 and C/S373 in (SER)-MBD4, and C12, C15, and K60 in Atox1). The QM region here is treated at the Density Functional Theory (DFT) level with the BLYP exchange-correlation functional [72,73] by employing a dual Gaussian-type/plane waves basis set (GPW) [74]. In particular, we used a double-ζ (MOLOPT) basis set [75] along with an auxiliary PW basis set with a density cutoff of 400 Ry and Goedecker–Teter–Hutter (GTH) pseudopotentials [76,77]. This level of theory has often been used in successful QM/MM MD simulations of biomolecules [53,70,78,79,80,81]. The dangling bonds between the QM and MM regions were saturated by using capping hydrogen atoms. All QM/MM MD simulations were performed by using an integration time step of 0.5 fs in the NVT ensemble. All systems were initially optimized, heated to 300 K in 2 ps, and equilibrated at 300 K without constraints for 5 ps by using a Nosé–Hoover thermostat.

During the QM/MM MD simulations, we observed that the Cu(I) coordination changed from tetrahedral (as in the crystal structure) to a linear bi-coordination. Therefore, we modified the force field parameters for classical MD (by changing the reference bond lengths and angle values of the Cu(I) coordinating residues, whereas the spring constants were kept at the same values as in ref [63]) in order to match the QM/MM MD geometry. Using these parameters, we performed additional 200 ns-long cMD simulations for all systems starting from a representative snapshot taken from the QM/MM MD simulations. Finally, we performed additional 5-ps-long QM/MM MD simulations on the highest populated clusters from the Cu(I)-bound Atox1-(SER)-MBD3/4 heterodimer and WT/SER-CueR systems in order to further and more accurately relax the structure of the systems.

## 5. Conclusions

This research reveals that Cu(I) ions can bind to proteins regulating copper homeostasis even in the presence of serine mutations. Nevertheless, cysteine-to-serine mutations induced the formation of non-functional proteins in the presence of Cu(I) ions, since they may have affected the global structure of the hosting proteins, possibly leading to their instability, and they increased the aggregation propensity. In addition, these mutations also affected the interaction between proteins in the copper transport cycle, and counteracted the proton delivery necessary for a fast Cu(I) exchange among them. 

## Abbreviations

Cys	Cysteine
Ser	Serine
WT	Wild type
CD	Circular dichroism
EPR	Electron paramagnetic resonance
CW-EPR	Continuous wave electron paramagnetic resonance
RT	Room temperature
O.N.	Overnight
MBD	Metal binding domain
NMR	Nuclear magnetic resonance
sm-FRET	Single molecule Förster energy transfer
sl	Spin-labeled
GA	Glutaraldehyde
MTSSL	Methanesulfonothiolate
EXAFS	Extended X-ray absorption structure
MD	Molecular dynamics
cMD	Classical molecular dynamics
QM/MM	Quantum Quantum-classical MD

## Appendix A

**Table A1:** Occurrence (%) of important hydrogen (H)-bonds between Atox1 and MBD4 during the 200 ns of cMD simulations. Occurrence is calculated as a percentage of the number of frames the H-bond is present with respect to the frames of the whole trajectory.

System	MBD4_CYS_apo	MBD4_CYS	MBD4_CYS_depr
H-Bond			
Lys60–Thr369 ^‡^ (NZ-HZ*^…^OG)	18.0	/	/
Lys60 – Ser376 ^‡^ (NZ-HZ*^…^OG)	/	/	60.9
Cys370 – Cys373 (O^…^H-N)	11.6	/	/

^‡^ Inter-protein hydrogen bond.

**Table A2:** Occurrence (%) of important H-bonds between Atox1 and MBD3 during the 200 ns of cMD simulations. Occurrence is calculated as a percentage of the number of frames the H-bond is present with respect to the frames of the whole trajectory.

System	MBD3_CYS_apo	MBD3_CYS	MBD3_CYS_depr
H-Bond			
Lys60–His267 ^‡^ (NZ-HZ*^…^O)	18.3	/	/
Lys60–Ser270 ^‡^ (NZ-HZ*^…^OG)	/	/	11.4
Lys60–Cys271 ^‡^ (NZ-HZ*^…^SG)	/	/	32.3
Lys60–Glu277 ^‡^ (NZ-HZ*^…^OE1/2)	/	50.7	/
Cys268–Cys271 (O^…^H-N)	14.7	/	/

^‡^ Inter-protein hydrogen bond.

**Table A3:** Occurrence (%) of important H-bonds between Atox1 and SER-MBD4 during the 200 ns of cMD simulations. Occurrence is calculated as a percentage of the number of frames the H-bond is present with respect to the frames of the whole trajectory.

System	MBD4_SER_apo	MBD4_SER	MBD4_SER_depr
H-Bond			
Cys12 – Thr369 ^‡^ (SG^…^HG-OG)	/	/	20.7
Cys15 – Ser373 ^‡^ (SG^…^HG-OG)	/	16.7	/
Lys60 – Thr369 ^‡^ (NZ-HZ*^…^OG)	23.4	/	/
Ser370 – Ser373 (O^…^H-N) (O^…^HG-OG) (N-H^…^OG) (OG^…^H-N)	48.2 37.5 / /	38.3 17.8 / /	/ / 70.3 22.3

^‡^ Inter-protein hydrogen bond.

**Table A4:** Occurrence (%) of important H-bonds between Atox1 and SER-MBD3 during the 200 ns of cMD simulations. Occurrence is calculated as a percentage of the number of frames the H-bond is present with respect to the frames of the whole trajectory.

System	MBD3_SER_apo	MBD3_SER	MBD3_SER_depr
H-Bond			
Cys12–His267 ^‡^ (SG^…^HE2-NE2)	/	/	54.1
Cys15–Ser270 ^‡^ (SG^…^HG-OG)	/	63.4	/
Cys15–Asn322 ^‡^ (SG^…^HD22-ND2)	/	/	15.1
Lys60–Gln281 ^‡^ (NZ-HZ*^…^OE1)	27.6	/	/
Ser268–Ser271 (O^…^H-N) (O^…^HG-OG)	/ /	55.9 86.0	/ /
Ser271–Thr11 ^‡^ (OG^…^HG-OG)	/	/	72.4

^‡^ Inter-protein hydrogen bond.

**Table A5:** Average distances (Å) between Cu(I) and O/S and angle (°) between them as obtained from QM/MM MS simulations and as used in cMD simulations (in parentheses).

System	WT_CueR (5.0 ps)	CueR_C112S_C120S (5.0 ps)	CueR_C120S (1.5 ps)
d(Cu–S/O@C112/C112S)	2.18 ± 0.05 (2.20)	1.88 ± 0.04 Å (1.87 )	2.19 ± 0.06 Å (2.20 )
d(Cu–S/O@C120/C120S)	2.17 ± 0.06 Å (2.20 )	1.87 ± 0.06 Å (1.87 )	1.83 ± 0.03 Å (1.87)
angle(S/O@C112/C112S–Cu–S/O@C120/C120S)	169.8 ± 5.2 (170.0)	170.4 ± 5.3 (170.0)	167.7 ± 6.1 (170.0)

**Table A6:** Relative population of the 5 highest populated clusters (%) for each CueR system as extracted from cMD trajectories.

Cluster No.	1	2	3	4	5
System					
WT_CueR	49.8	47.5	1.8	0.6	0.3
CueR_C112S_C120S	76.4	16.5	4.0	2.0	1.2
CueR_C120S	72.9	12.4	11.6	2.4	0.8

**Table A7:** Occurrence (%) of important H-bonds between WT and mutant (C112S_C120S and C120S) CueR protein isoforms during the 200 ns of cMD simulations. Occurrence is calculated as a percentage of the number of frames the H-bond is present with respect to the frames of the whole trajectory.

System	WT_CueR		CueR_C112S_C120S		CueR_C120S	
H-Bond						
Cys/Ser112–Lys81 ^‡^ (O^…^HZ*-NZ) (SG/OG^…^HZ*-NZ)	/ /	/ 17.3	27.0 /	38.9 /	16.6 66.7	/ /
Cys/Ser112–Leu108 (N-H^…^O)	/	/	11.6	4.9	/	/
Cys/Ser112–Ala109 (N-H^…^O)	/	/	33.9	44.8	30.0	/
Cys/Ser112–Asn110 (O^…^HD2*-ND2) (N-H^…^OD1)	14.2 56.5	/ /	/ /	/ /	/ /	/ /
Cys/Ser112–Gly114 (SG/OG^…^H-N)	/	17.9	/	/	/	/
Cys/Ser120–Arg75 ^‡^ (SG/OG^…^HH1*-NH1) (SG/OG^…^HH2*-NH2)	/ /	74.8% 83.5%	/ /	/ /		/ /
Cys/Ser120–Lys81 ^‡^ (SG/OG…HZ*-NZ)	/	/	/	/	/	13.7
Cys/Ser120–Ser117 (N-H^…^O) (N-H^…^OG)	/ /	/ /	/ /	/ /	11.9 25.6	/ 14.7
Cys/Ser120–Ile122 (O^…^H-N) (SG/OG^…^H-N)	/ /	20.9 /	/ 27.2	/ 21.1	/ /	/ /
Cys/Ser120–Ile123 (O^…^H-N)	/	14.6	/	/	43.3	/
Cys/Ser120–Glu124 (O^…^H-N)	/	/	47.0	47.0	/	/

^‡^ Inter-protein hydrogen bond.
